# Characteristics of childhood allergic diseases in outpatient and emergency departments in Shanghai, China, 2016–2018: a multicenter, retrospective study

**DOI:** 10.1186/s12887-021-02880-0

**Published:** 2021-09-17

**Authors:** Yuanyuan Qi, Peng Shi, Renjie Chen, Yufeng Zhou, Lijuan Liu, Jianguo Hong, Lanfang Cao, Yanming Lu, Xiaoyan Dong, Jing Li, Yu Shi, Min Xia, Bo Ding, Liling Qian, Libo Wang, Wenhao Zhou, Yonghao Gui, Xiaobo Zhang

**Affiliations:** 1 Department of Respiratory Medicine, Children’s Hospital of Fudan University, National Children’s Medical Center, Shanghai, 201102 China; 2 Department of Data Management and Statistics, Children’s Hospital of Fudan University, Shanghai, China; 3 School of Public Health, Key Lab of Public Health Safety of the Ministry of Education and NHC Key Laboratory of Health Technology Assessment, Fudan University, Shanghai, 200032 China; 4 Institute of Pediatrics, Children’s Hospital of Fudan University, Shanghai, 201102 China; 5 Institutes of Biomedical Sciences, Fudan University, Shanghai, 200032 China; 6 Department of Pediatrics, Shanghai General Hospital, Shanghai Jiaotong University, Shanghai, 200080 China; 7 Department of Pediatrics, Renji Hospital, Shanghai Jiao Tong University, Shanghai, 200127 China; 8 Department of Pediatrics, South Campus, Renji Hospital, Shanghai Jiao Tong University, Shanghai, 201112 China; 9 Department of Respiratory Medicine, Children’s Hospital of Shanghai Jiaotong University, Shanghai, 200040 China; 10 Big Data Product Department, Wonders Information Co. Ltd., Shanghai, China; 11 Department of Neonatology, Children’s Hospital of Fudan University, National Children’s Medical Center, Shanghai, 201102 China; 12 Cardiovascular Center, Children’s Hospital of Fudan University, National Children’s Medical Center, Shanghai, 201102 China

**Keywords:** Allergic diseases, Asthma, Allergic rhinitis, Comorbidity, Outpatient visit

## Abstract

**Background:**

The prevalence of allergic diseases (ADs), such as asthma and allergic rhinitis (AR), is increasing worldwide in both adults and children. Although ADs are common and frequently coexist in outpatient care, city-level data regarding the characteristics of childhood AD remain limited in China. This study aimed to assess the profile and characteristics of ADs in the city of Shanghai.

**Methods:**

A multicenter retrospective study was designed to collect routine administrative data from outpatient and emergency departments from 66 hospitals in Shanghai, China, from 2016 to 2018. Children with asthma, AR, allergic conjunctivitis (AC), and allergic skin diseases were investigated. Demographic characteristics, patients visit pattern, spectrum of diagnosis, and comorbidities were analyzed.

**Results:**

A total of 2,376,150 outpatient and emergency visits for ADs were included in the period from 2016 to 2018. Allergic skin diseases accounted for 38.9%, followed by asthma (34.8%), AR (22.9%), and AC (3.3%), with a male predominance in all four diseases. Asthma and allergic skin diseases were most frequent in the 1 to < 4 years of age group, while AR and AC were more common in the 4 to < 7 years of age group. Asthma accounted for the greatest number of annual and emergency visits. The most frequent comorbidity of asthma was lower respiratory tract infection (LRTI) (49.3%), followed by AR (20.5%) and upper respiratory tract infection (14.1%). The most common comorbidities of AR were otitis media (23.4%), adenoid hypertrophy/obstructive sleep apnea (22.1%), followed by LRTI (12.1%), asthma (9.4%) and chronic pharyngitis (8.9%).

**Conclusions:**

Asthma and allergic skin diseases were the most common ADs in outpatient and emergency departments in the study period. Respiratory tract infection was the most common comorbidity of asthma in children. More attention should be devoted to the treatment of comorbidities to improve childhood AD outcomes with a better understanding of the characteristics of ADs in outpatient care.

**Supplementary Information:**

The online version contains supplementary material available at 10.1186/s12887-021-02880-0.

## Background

Allergic diseases (ADs) represent one of the most common types of respiratory diseases globally, not only in adults, but also in children and adolescents [1]. Over the past decades, the prevalence of ADs has increased worldwide [2, 3]. The complexity and severity of ADs continue to increase, especially in children, resulting in substantial healthcare expenditures and a high burden on patient’ quality of life [4].

The prevalence of ADs varies greatly according to geographical region. Huang et al. [5] reported a prevalence of 10.2% for asthma, 54.1% for rhinitis, and 22.5% for eczema in Shanghai, China. A retrospective study conducted in a tertiary pediatric hospital in Shanghai reported that asthma and rhinitis accounted for 20% of respiratory outpatient visits [6]. ADs are often associated with various comorbidities, which have a significant impact on health outcomes [7–10]. Although ADs are common and frequently coexist in outpatient care, data regarding the characteristics of childhood AD have remained limited in China for a prolonged period. Understanding the profile and characteristics of ADs can provide insight into local health policies and guide clinical practice.

The primary objective of the present study was to describe the characteristics of ADs (asthma, allergic rhinitis [AR], allergic skin diseases, and allergic conjunctivitis [AC]) in selected outpatient and emergency departments in Shanghai, China, and comorbidities of ADs.

## Methods

### Study design

A multicenter, retrospective study was designed to collect routine administrative data from two children hospitals and 64 general hospitals with pediatric clinics that provide the comprehensive services for children in Shanghai, China. Data regarding visits from children ≤18 years of age, who were diagnosed with ADs, were collected from the hospital information system (HIS) of the outpatient department and emergency department (ED) between January 1, 2016, and December 31, 2018. Demographic information, such as age, sex, date of birth, medical records of date of visit, and diagnosis, were included. Patient age was calculated as the difference between birth date and visit date, and classified as < 1, 1 to < 4, 4 to < 7, 7 to < 12, and 12 to ≤18 years. The study protocol was approved by the Research Ethics Committee of the Children’s Hospital affiliated to Fudan University (Shanghai, China; NO. 2018–215). All protocols were performed in accordance with the relevant guidelines and regulations.

### Definitions of conditions

The most common allergic disorders among children were investigated, including asthma, AR, allergic skin diseases, and AC. Allergic skin diseases in the study included atopic dermatitis, allergic contact dermatitis, urticaria, angioedema, and drug eruption. *The International Classification of Diseases, 10th Revision* codes (ICD-10), and detailed codes for each disease are shown in supplementary Table 1.

Cough variant asthma (CVA) was diagnosed according to the following criteria: clinical history of a persistent cough (≥ 4 weeks) and airway high reaction, but no wheezing or dyspnea, in contrast to classic asthma, anti-asthma drugs effective against their coughs, excluding other diseases that caused chronic cough.

Severe asthma attack was diagnosed according to criteria from the “Guideline for the diagnosis and optimal management of asthma in children (2016)” in China, which is characterized by respiratory distress, use of accessory muscles, tachypnea, tachycardia, difficulty with speaking in full sentences, decreased level of consciousness, oxygen saturation 90%, and peak expiratory flow ≤50% of predicted for children ≥6 years of age, and was characterized by respiratory distress, decreased level of consciousness, oxygen saturation ≤ 92%, difficulty speaking in full sentences, cyanosis, or quiet chest for children < 6 years of age.

Status asthmaticus was defined as a prolonged and severe asthma attack that did not respond to standard or optimal treatment.

Atopic dermatitis is a chronic inflammatory skin disease characterized by eczematous lesions and is associated with elevated serum immunoglobulin (Ig) E levels, and tissue and blood eosinophilia.

Diagnostic tests, including peripheral eosinophils, serum total and specific IgE, and/or skin prick tests, were performed by the healthcare providers according to clinical conditions.

### Statistical analyses

Python was used for data processing and SPSS version 22.0 (IBM Corporation, Armonk, NY, USA) for data analysis. For descriptive analyses, numbers and percentages (%) were used for qualitative variables. The chi-squared test was used to test differences in proportions between the groups. The average annual growth rate (AAGR) was calculated in terms of the geometric growth, expressed as (X_*n*_/X_*0*_)^(1/*n*)^-1 (where X_*0*_ is the value of the baseline and X_*n*_ is the value of the *n*th year).

The missing data imputation were not applied in this study for the administrative data when the missing rate is low (Supplementary Table 2).

Differences with a two-tailed *P* value ≤0.05 was considered to be statistically significant. Due to the large sample sizes in this study, effect sizes were more meaningful than *P* values in the interpretation of the results.

## Results

### Overview of visits from patients with ADs

There were a total of 2,376,150 outpatient and emergency visits for ADs in the 66 hospitals from 2016 to 2018 in Shanghai. The total number of visits increased from 657,217 in 2016 to 917,565 in 2018, corresponding to an AAGR of 13.2%. Among ADs, allergic skin diseases accounted for 38.9%, followed by asthma (34.8%), AR (22.9%), and AC (3.4%) (Table 1).

**Table 1:** Overview of visits from patients with allergic diseases in Shanghai, China, 2016 to 2018

Diseases	2016	2017	2018	Total
Asthma, *n* (%)	267,091 (40.6)	260,887 (32.6)	298,939 (32.6)	826,917 (34.8)
AR, *n* (%)	120,196 (18.3)	179,142 (22.3)	243,873 (26.5)	543,211 (22.9)
Allergic skin diseases, *n* (%)	249,596 (38.0)	333,284 (41.6)	341,857 (37.3)	924,737 (38.9)
AC, *n* (%)	20,334 (3.1)	28,055 (3.5)	32,896 (3.6)	81,285 (3.4)
Total	657,217	801,368	917,565	2,376,150

### Demographic characteristics of patients with ADs

The proportions of sex, payer type, and age groups were compared among the four disease groups (Table 2). Male predominance was found in all four diseases. A lower male-to-female ratio was observed in allergic skin diseases than in asthma, AR, and AC (1.2 for allergic skin diseases, 1.9 for asthma, 1.7 for AR, and 1.8 for AC, *P* < 0.001).

**Table 2:** Comparison of demographic characteristics of patients with allergic diseases

Characteristic	Asthma	AR	Allergic skin diseases	AC	*P* value
Gender					< 0.001
Male	541,755 (65.5)	342,530 (63.1)	513,255 (55.5)	52,600 (64.7)	
Female	284,987 (34.5)	198,982 (36.6)	411,428 (44.5)	28,679 (35.3)	
Age range, years					< 0.001
< 1	78,989 (9.6)	14,373 (2.6)	241,955 (26.2)	2717 (3.3)	
1 ~ < 4	313,363 (37.9)	133,686 (24.6)	309,163 (33.4)	22,697 (27.9)	
4 ~ < 7	268,172 (32.4)	207,631 (38.2)	170,555 (18.4)	30,045 (37.0)	
7 ~ < 12	134,371 (16.2)	144,785 (26.7)	137,207 (14.8)	22,881 (28.1)	
12 ~ ≤18	32,022 (3.9)	42,736 (7.9)	65,857 (7.1)	2945 (3.6)	
Payer type					< 0.001
Medical insurance	539,264 (65.2)	349,116 (64.3)	492,112 (53.2)	55,400 (68.2)	
Self-finance	284,987 (34.8)	194,095 (36.7)	432,625 (46.8)	25,885 (31.8)	

For payer type, the proportion of children with government insurance was lower in allergic skin diseases (53.2%) than in the other diseases (65.2% for asthma, 64.3% for AR, and 68.2% for AC, *P* < 0.001).

Regarding the age distribution of ADs, in patients with asthma, the 1 to < 4 years’ age group (37.9%) was the most common, followed by the 4 to < 7 (32.4%), 7 to < 12 (16.2%), < 1 (9.6%), and 12 to ≤18 (3.9%) years’ age group. The 1 to < 4 years’ age group (33.4%) was most common in allergic skin diseases, followed by < 1 (26.2%), 4 to < 7 (18.4%), and 7 to < 12 (14.8%) years’ age groups. The 4 to < 7 years’ age group was most common in AR and AC, followed by the 7 to < 12, 1 to < 4, and < 1 years’ age groups.

### Visit patterns of patients with ADs

The proportion of ED visits was highest for asthma (19.8%), followed by allergic skin diseases (8.1%), AR (4.1%), and AC (2.1%). Demographic characteristics between outpatient visits and ED visits were compared (Supplementary Table 3). The number of visits by different age groups, genders, and payer types in the ED visits were found to be of a similar order as the outpatient department visits. For asthma, the proportion of boys was higher in ED visits than in outpatient visits. In contrast, the proportion of girls was higher in ED visits than in outpatient visits for AR and allergic skin diseases. The ED visit ratio for asthma was highest in the 7 to < 12 years’ age group, followed by the 12 to ≤18, 1 to < 4 years’ age group, the < 1 years’ age group had the lowest ED visit ratio. The ratio of ED visits increased with age in the AR. The proportion of those with medical insurance was higher in ED visits than in outpatient visits for asthma, AR, and allergic skin diseases.

**Fig. 1:**
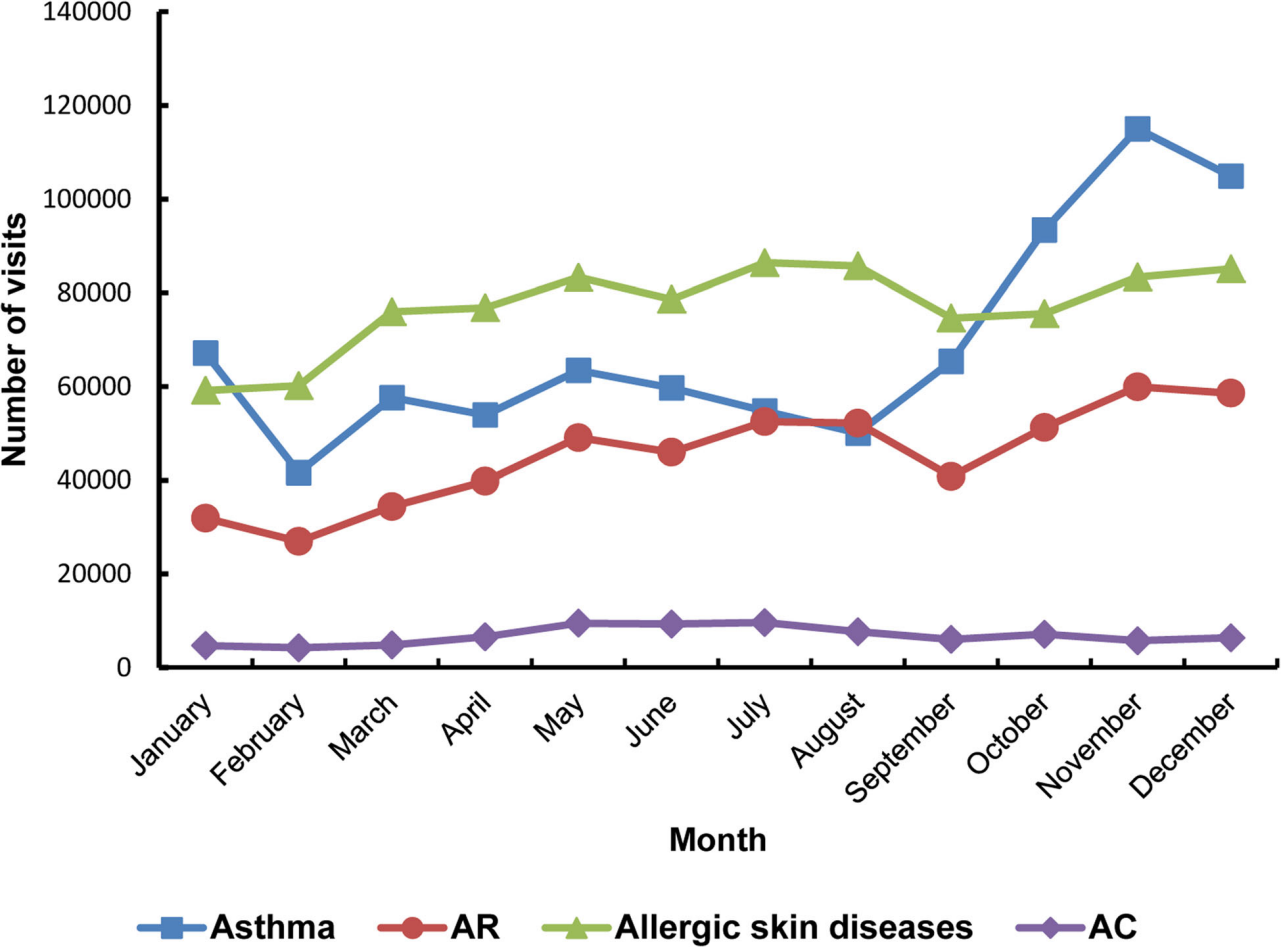
Seasonal variations in the number of visits for allergic diseases. AR, allergic rhinitis; AC, allergic conjunctivitis

Seasonal visits according to month are presented in Fig. 1. The number of visits for asthma was lowest in February, increasing slightly in the spring, reduced in the summer, increased rapidly in September, and then reached a peak in November and December. Visits for AR were high in November and December and low from January to March. Visits for allergic skin diseases were high in July and August and low in January and February. Visits for AC were stable throughout the whole year, with a slight increase from May to July.

### Spectrum and comorbidities of ADs

Among the visits for asthma, the proportion of CVA was 9.5%, with an increase from 2016 to 2018 (Table 3). Asthma complicating respiratory infection accounted for 3.7% of total asthma visits, and with a decrease observed from 2016 to 2018. A total of 2636 (0.3%) visits were for severe asthma attacks, and 62 patients were diagnosed with status asthmaticus.

**Table 3:** Spectrum of diagnosis of asthma and allergic skin diseases

Characteristic	2016	2017	2018	Total
Asthma				
Asthma alone	237,480 (88.9)	233,497 (89.5)	243,975 (81.6)	714,952 (86.5)
Asthma complicated by respiratory infection	14,361 (5.4)	7790 (3.0)	8275 (2.8)	30,729 (3.7)
Severe asthma attack	570 (0.2)	781 (0.3)	1588 (0.5)	2636 (0.3)
Status asthmaticus	24 (0.009)	26 (0.010)	12 (0.004)	62 (0.007)
Cough variant asthma	14,620 (5.5)	18,736 (7.2)	45,047 (15.7)	78,403 (9.5)
Allergic skin disease				
Atopic dermatitis	176,885 (70.9)	242,312 (72.7)	235,712 (69.0)	654,909 (70.8)
Acute urticaria	68,853 (27.6)	84,410 (25.3)	96,826 (28.3)	250,089 (27.0)
Chronic urticaria	2406 (0.96)	2901 (0.870)	2974 (0.870)	8281 (0.895)
Contact dermatitis	780 (0.313)	1861 (0.558)	3125 (0.914)	5766 (0.624)
Angioedema	552 (0.221)	1465 (0.440)	2665 (0.780)	4682 (0.506)
Photosensitive dermatitis	114 (0.046)	196 (0.059)	347 (0.102)	657 (0.071)
Drug Eruption	6 (0.002)	139 (0.042)	208 (0.061)	353 (0.038)

Furthermore, patient characteristics were compared between those with classic asthma and CVA. The proportion of ED visits was lower for CVA (9.7%) than for classic asthma (21.3%). The number of visits was highest in 4 to < 7 years’ age group (46.9%) for CVA and in the 1 to < 4 years’ group for classic asthma. The male-to-female ratio was lower in patients with CVA than in those with classic asthma.

Atopic dermatitis (70.8%) was the most common allergic skin disease, followed by acute urticaria (27%). Other allergic skin diseases included chronic urticaria (0.895%), contact dermatitis (0.624%), and angioedema (0.506%). The proportion of drug eruptions was found to be 0.038%. For atopic dermatitis, < 1-year-old group (32.8%) was the most common, followed by 1 to < 4 (30.4%), 4 to < 7 (16.7%), 7 to < 12 (13.9%), and 12 to ≤18 (6.2%) years’ age groups.

**Fig. 2:**
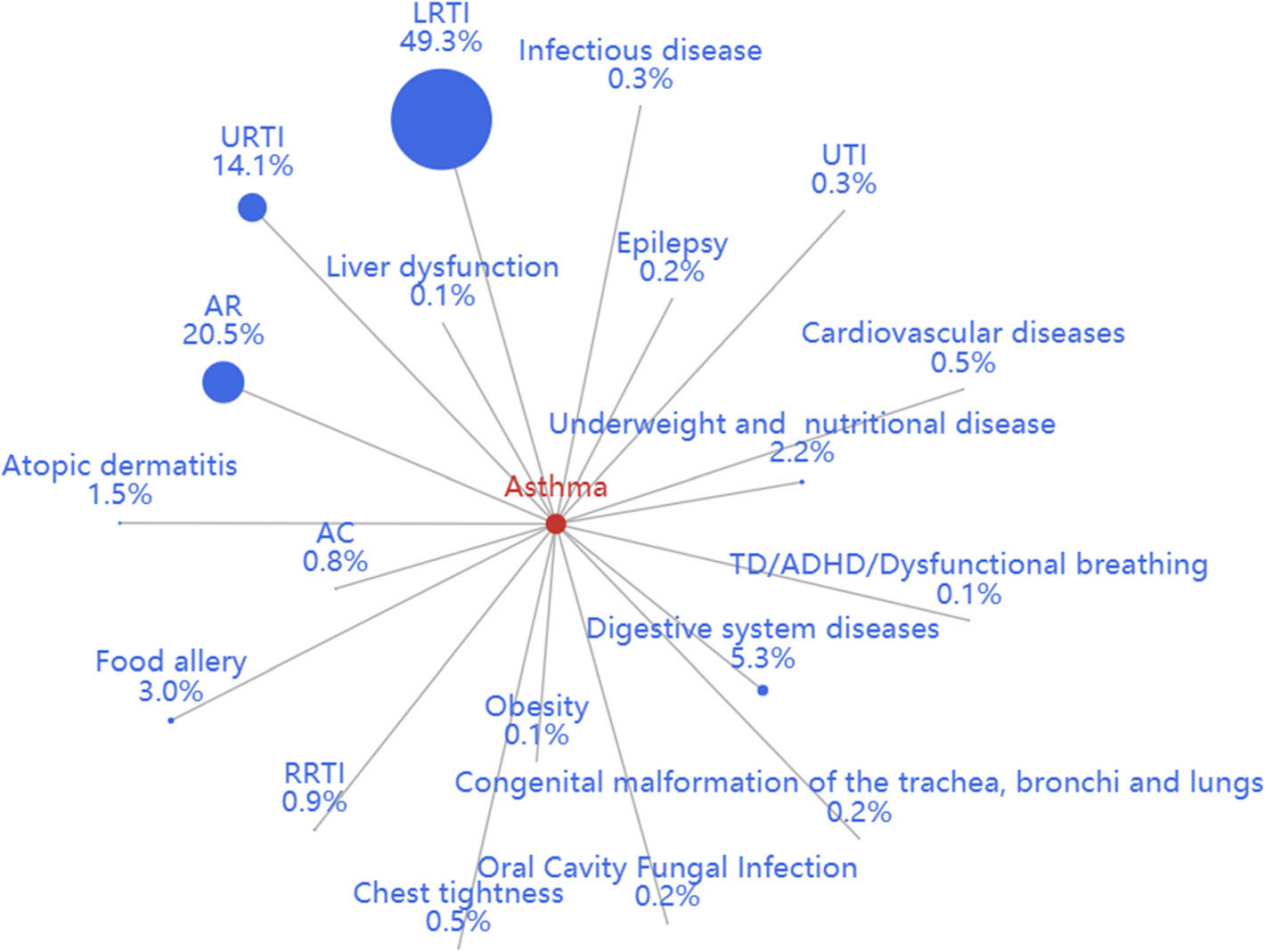
The constituent ratio of comorbidities in asthma. Abbreviations: RRTI, recurrent respiratory tract infection; AC, allergic conjunctivitis; AR, allergic rhinitis; TD, tic disorder; ADHD, attention-deficit hyperactivity disorder; LRTI, lower respiratory tract infection; URTI, upper respiratory tract infection; UTI, urinary tract infection

The comorbidities of asthma and CVA were analyzed separately. Of the 747,597 visits for asthma in the 3 years, 37,693 (5.0%) had comorbidities (Fig. 2). The most common comorbidities of asthma were lower respiratory tract infection (LRTI) (49.3%), AR (20.5%), and upper respiratory tract infection (URTI) (14.1%). Other allergic comorbidities included food allergies or others (2.98%), allergic skin diseases (1.51%), and AC (0.75%). Moreover, a small percentage of patients also had co-conditions such as epilepsy/seizure disorder (0.18%) and tic disorder (TD)/ attention deficit hyperactivity disorder (ADHD) (0.14%).

**Fig. 3:**
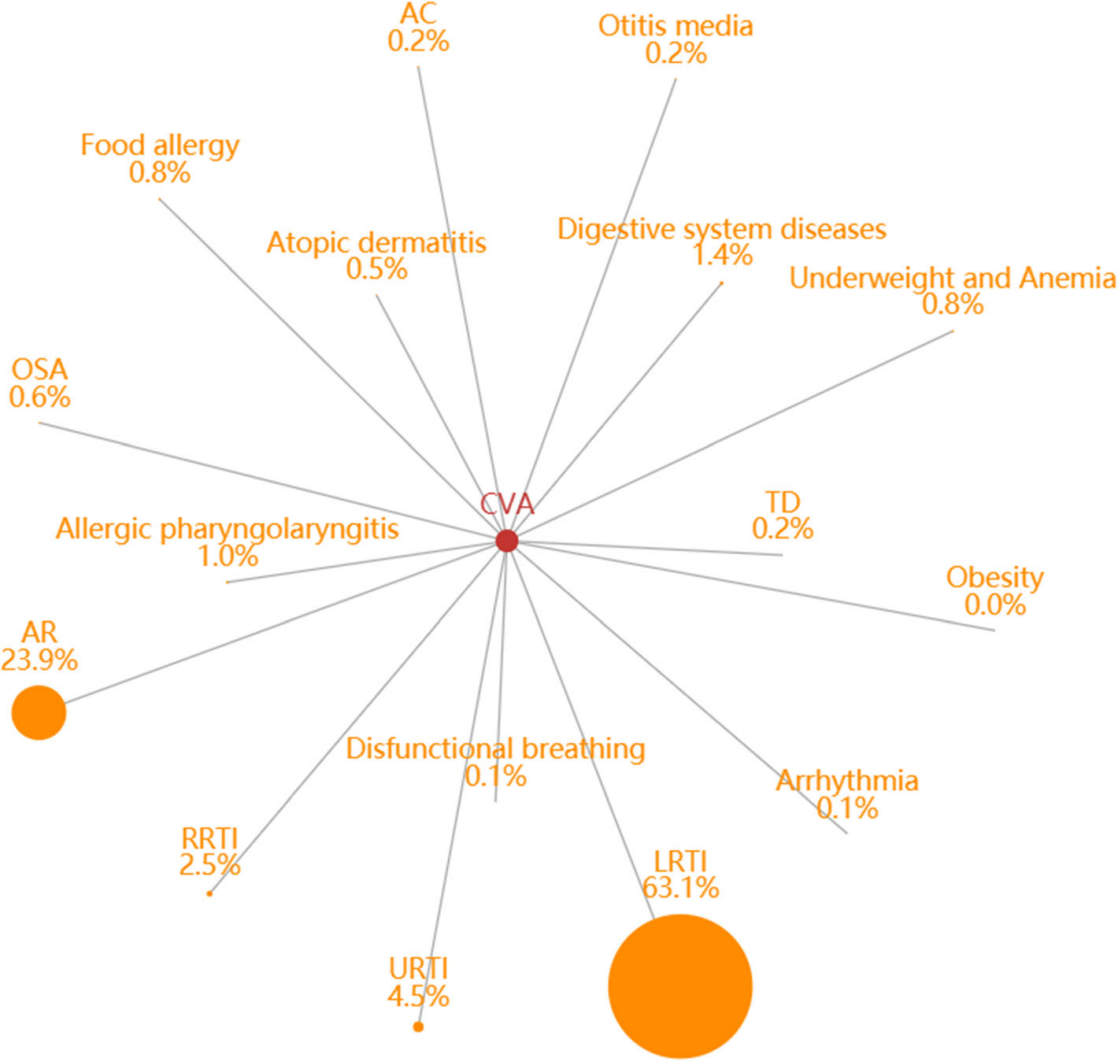
The constituent ratio of comorbidities in cough variant asthma (CVA). Abbreviations: RRTI, recurrent respiratory tract infection; AC, allergic conjunctivitis; AR, allergic rhinitis; TD, tic disorder; LRTI, lower respiratory tract infection; URTI, upper respiratory tract infection; OSA, obstructive sleep apnea

Patients with CVA had a higher comorbidity rate than those with classic asthma. Of the all 78,403 visits for CVA, 22,281 (28.4%) had coexisting conditions (Fig. 3). Compared with asthma, CVA had a higher proportion of coexisting LRTIs (63.1%) and a lower proportion of URTIs (4.54%). AR was the second most common comorbidity of CVA. Other allergic comorbidities included allergic pharyngolaryngitis (0.96%), food allergy or others (0.84%), obstructive sleep apnea (OSA) (0.62%), allergic skin diseases (0.46%), and AC (0.25%).

**Fig. 4:**
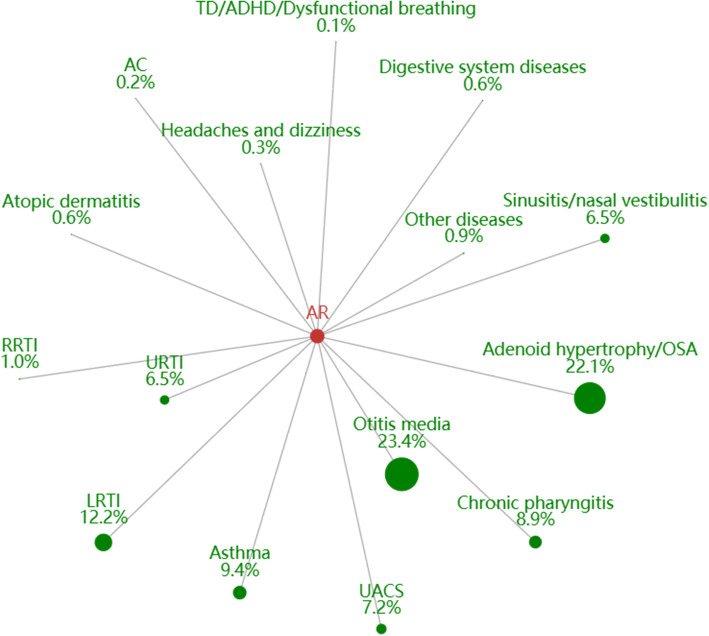
The constituent ratio of comorbidities in AR. Abbreviations: RRTI, recurrent respiratory tract infection; AC, allergic conjunctivitis; AR, allergic rhinitis; TD, tic disorder; ADHD, attention-deficit hyperactivity disorder; LRTI, lower respiratory tract infection; URTI, upper respiratory tract infection; OSA, obstructive sleep apnea; UACS, upper airway cough syndrome

Of the 543,211 allergic rhinitis visits for 3 years, 78,266 (14.4%) had any concomitant diseases (Fig. 4). The most common comorbidities were otitis media (23.4%), adenoid hypertrophy/OSA (22.1%), followed by LRTI (12.1%), asthma (9.4%), chronic pharyngitis (8.9%), upper airway cough syndrome (UACS) (7.2%) and rhinosinusitis (6.5%). Other coexist allergic conditions included allergic skin diseases (0.56%), allergic conjunctivitis (0.25%).

## Discussion

We reported a multicenter retrospective study to describe the characteristics of asthma, AR, allergic skin diseases and AC in pediatric outpatient and emergency settings in Shanghai, China.

The global prevalence of AD has increased dramatically in recent decades. However, the prevalence and spectrum of AD vary according to region and race. Alemayehu et al. and David et al. reported that the rank order of common ADs was asthma, AR, atopic dermatitis and AC [11, 12]. A study from Korea reported that the most common AD was asthma, followed by atopic dermatitis, AR, and AC [13]. In the present study, allergic skin diseases were the most frequent, followed by asthma and AR. The different prevalences of ADs among areas may be attributed to differences in genetic background and environmental factors. We did not include the data on food allergy and drug hypersensitivity due to the lack of pediatric allergy clinics; nevertheless, further research is needed.

In our study, the proportion of AR among ADs increased significantly from 2016 to 2018. There may be several reasons for this finding, the first of which is that outdoor and indoor environmental pollution may be associated with the increased prevalence of AR. Second, due to intensive training for pediatricians in asthma and AR, more patients were diagnosed in a timely manner.

In our study, there was only a small proportion of patients with AC, and was lower than that reported in other studies [11–13]. There may be several reasons for this difference. First, the majority of patients experienced few episodes of mild and intermittent symptoms. Second, only a few of the medical settings had ophthalmology outpatient departments for children. More attention should be devoted to the diagnosis and treatment of AC in the future.

It is well known that ADs have a natural history, and the incidence of specific ADs varies according to age. A study involving a Swedish primary care asthma population reported that the number of asthma patients was significantly higher in the 7–14 years’ group than in the < 7 years’ group [14]. An epidemiological study from the United States documented an increasing trend in the prevalence of asthma with age [12]. However, a recent meta-analysis reported the highest prevalence in children 5–9 years of age in 2010 in China [15]. In the present study, the number of visits for asthma was highest in the 1 to < 4 and 4 to < 7 years’ group. A possible explanation for this inconsistency is the high incidence of respiratory infection in the 1 to < 4 years’ group. The prevalence of atopic dermatitis is believed to decrease with increasing age [16]. In our study, atopic dermatitis was observed most commonly in the < 1 and 1 to < 4 years’ groups, which was similar to previous reports [17, 18].

Our results suggest a higher prevalence of ADs in boys compared to girls. This is consistent with previous studies that reported a male predominance [19]. The overall percentage of medical insurance in allergic skin diseases group is lower than other allergic disease groups. This may be partly due to the delayed medical insurance procedures in infants by their parents. However, other factors contributing to this discrepancy, e.g. economic status, environmental factors, needs further investigation.

Seasonal variations in AD have been of great interest in clinical practice. However, there are few studies with large-scale epidemiological data. In the present study, clear seasonality was found in the four ADs, with evident differences among them.

Regarding asthma, many factors may influence the risk for visit for asthma [20]. It has been reported that asthma peaked in winter and spring and was negatively correlated with both temperature and humidity [21]. In this study, the increased visits for asthma in autumn and winter could be mainly caused by increased viral infection or cold temperatures. However, there was a sharp decrease in the number of visits in February, which may be due to the winter vacation when cross infections decreased.

Two types of seasonal patterns (“summer” type and “winter” type) of atopic dermatitis have been reported [22]. Noh et al. reported a negative association between atopic dermatitis symptoms and temperature [23]. Fleischer et al. found that increased temperatures predicted increased atopic dermatitis office visits [24]. The relative higher temperature and humidity in summer may contribute to the higher visits of allergic skin diseases in the present study.

Similar to a previous study, more visits for AR were observed in colder months in the present study. However, there were also high numbers of visits for AR from May to August, which may be associated with a corresponding increase in aeroallergens, such as pollen [25]. The outpatient visits for AC increased from May to July, which was consistent with a previously literature [26]. This may be explained by the increase of temperature and pollen.

Studies have also found that air pollution have adverse impact on ADs [27–29]. Further research is needed to ascertain the specific factors that explain the different seasonal variations of allergic diseases. Moreover, the visit trend may be associated with the total visit characteristics of outpatient and emergency departments, and further research should be conducted to evaluate its influence.

In the present study, we found that boys had a higher ED visit ratio than girls with asthma, which is consistent with other reports [30]. In this study, the ED ratios were the lowest in the < 1 year group and increased with increasing age. There are conflicting reports in the literature regarding the influence of age on ED visits for asthma. Wasilewski et al. [31] reported that younger children exhibited a higher risk of ED visits. However, Tolomeo et al. [32] found that the risk of EV visits increase with age, which is consistent with our findings. This can be partly explained by different triggers of asthma exacerbations. Another possible explanation is that school-aged children are more likely to choose ED visit after school to avoid missing the school lessons.

CVA is easily overlooked and clinically misdiagnosed. In this study, we compared the clinical features of classic asthma and CVA. CVA had a lower ED visit and a lower male ratio. This is consistent with previous reports that females were more frequently affected by CVA than classic asthma, although mechanisms of sex differences remain unclear. Studies have documented that CVA has a shorter duration of disease and higher forced expiratory volume in 1 s. Gao et al. [33] reported that patients with CVA experienced a shorter disease duration and higher lung function than patients with classic asthma. This may be the reason why CVA emergency visits were lower than those classic asthma. Patients with CVA had a higher proportion of ALRI than those with classic asthma. *Mycoplasma pneumoniae* infection has been reported to be related to the development of CVA. The pathogen in LRTI may be involved in CVA; however, the exact mechanism requires further research. We did not analyze the hospitalization rate and duration of hospitalization, and further research is needed to clarify these differences.

The findings that asthma and AR are associated with comorbid conditions have been previously demonstrated [34], and that comorbidities vary according to age group. LRTI was the most prevalent comorbidity in asthma in our study, whereas URTI was the most common comorbidity in all age group studies [14]. Gastroesophageal reflux disorder (GERD) is known to coexist with asthma [35]. However, the percentage of GERD in comorbidities was very low in the present study, suggesting that this comorbidity may be underestimated and undertreated in outpatient care for asthma. For AR, the most frequent comorbidities were otitis media and adenoid hypertrophy, while only a few percent of patients exhibited coexistent conjunctivitis and atopic dermatitis. It is noteworthy that AC was less common than AR in the present study than reported previously [36], partly because parents may have overlooked AC symptoms in their children. The association between neurobehavioral comorbidities and asthma or AR has been recently reported [37–40]. We also observed an association between TD/ADHD and ADs. The results indicated that more attention should be devoted to these co-conditions and should prompt further studies investigation the underlying mechanisms.

There were limitations to this study. The prevalence of ADs was not investigated in this study due to the lack of data for private medical institution visits and data of the catchment of hospitals. We did not include data regarding asthma and AR-related medication prescriptions, allergen immunotherapy, and the level of asthma control in asthma patients was not included in the present study. Therefore, further investigations should be conducted.

## Conclusion

In summary, allergic skin diseases and asthma were the two major diseases in outpatient and emergency visits for ADs in children in Shanghai. Respiratory tract infection was the most common comorbidity of asthma. More attention should be devoted to early diagnosis and comprehensive treatment of ADs in children.

## Supplementary Information

**Additional file 1: Supplementary Table 1.** The detailed ICD codes for diseases.

**Additional file 2: Supplementary Table 2.** Missing rate of gender and distribution comparison of age and payer type between missing group and non-missing group.

**Additional file 3: Supplementary Table 3.** Comparison of demographic characteristics between outpatient and ED visits.

## Data Availability

The data collected and analyzed during the current study are available from the corresponding author on reasonable request.

## Abbreviations

ADs: Allergic diseases
AR: Allergic rhinitis
AC: Allergic conjunctivitis
LRTI: Lower respiratory tract infection
OSA: Obstructive sleep apnea
HIS: Hospital information system
ED: Emergency department
ICD-10: Codes of International Classification of Disease, Revision 10
CVA: Cough variant asthma
TD: Tic disorder
ADHD: Attention deficit hyperactivity disorder
URTI: Upper respiratory tract infection
GERD: Gastroesophageal reflux disorder
RRTI: Recurrent respiratory tract infection
ADHD: Attention-deficit hyperactivity disorder
UACS: Upper airway cough syndrome
UTI: Urinary tract infection
