# Supplementary material for: Characteristics of childhood allergic diseases in outpatient and emergency departments in Shanghai, China, 2016–2018: a multicenter, retrospective study
Source: BMC Pediatr. 2021 Sep 17;21:409. doi: 10.1186/s12887-021-02880-0 (PMC8447671; doi:10.1186/s12887-021-02880-0)
Supplement: Supplementary file 1 — Additional file 1: Supplementary Table 1. The detailed ICD codes for diseases. [file 12887_2021_2880_MOESM1_ESM.pdf]

Supplementary Table 1. The detailed ICD codes for diseases

| NO | Diagnosis | ICD codes before 2017 | New ICD codes since 2018 |
|---|---|---|---|
| 1 | asthma | 'J45.000'，'J45.001'，'J45.002'，'J45.003'，'J45.004'，'J45.005'，'J45.006'，'J45.007'，'J45.100'，'J45.800'，'J45.900'，'J45.900A'，'J45.901'，'J45.902'，'J45.903'，'J45.904'，'J46.x00'， | J45.00000S01’,’ J45.00000S02’,’ J45.00000S03’,’ J45.00100S02’,’ J45.00200S01’,’ J45.00200S02’,’ J45.00300S01’,’ J45.0040S01’,’ J45.00500S01’,’ J45.00500S02’,’ J45.00600S01’,’ J45.00600S02’,’ J45.00600S03’, ‘J45.00600S04’,’ J45.00700S01’,’ J45.10000S01’,’ J45.10000S02’,’ J45.10000S03’,’ J45.10000S04’,’ J45.80000S01’,’ J45.90000S01’,’ J45.90000S02’,’ J45.90000S03’,’ J45.90000S04’,’ J45.90000S05’,’ J45.90000S06’,’ J45.90000S07’,’ J45.90000S08’,’ J45.90000S09’,’ J45.90000S0A’,’ J45.90000S0B’,’ J45.90000S0C’,’ J45.90000S0D’,’ J45.900A0S01’,’ J45.90100S01’,’ J45.90100S02’,’ J45.90100S03’,’ J45.90200S01’,’ J45.90200S02’,’ J45.90200S03’,’ J45.90300S01’,’ J45.90300S02’,’ J46.x0000S01’,’ J46.x0000S02’,’ J46.x0000S03’,’ J46.x0000S04’.’ J46.x0000S05’,’ J46.x0000S06’,’ J46.x0000S07’,’ |
| 2 | AR | 'J30.100'，'J30.101'，'J30.300'，'J30.400' | J30.10000S01’,’ J30.10000S02’,’ J30.10000S03’,’ J30.10100S01’,’ J30.10100S02’,’ J30.30000S01’,’ J30.30000S02’,’ J30.30000S03’,’ J30.40000S01’,’ J30.40000S02’,’ J30.40000S03’,’. |
| 3 | Allergic skin diseases | 'L20.802'，'L20.804'，'L20.900'，'L23.901'，'L25.900'，'L28.203'，'L30.203'，'L30.902'，'L50.000'，'L50.300'，'L50.301'，'L50.801'，'L50.802'，'L50.900'，'L56.300'，'L57.802'， | ‘L20.80200S01’,’ L20.80400S01’,’ L20.90000S01’,’ L20.90000S02’,’ L20.90000S03’,’ L21.90000S01’,’ L23.90100S01’,’ L23.90100S02’,’ L25.90000S01’,’ L25.90000S02’,’ L25.90000S03’,’ L25.90000S04’,’ L28.20300S01’,’ L30.20300S01’,’ L30.90200S01’,’ L30.90200S02’,’ L30.90200S03’,’ L30.90200S04’,’ L50.00000S01’,’ L50.30000S01’,’ L50.30100S01’,’ L50.80100S01 ’,’ L50.80200S01’,’ L50.90000S01’,’ L56.30000S01’,’ L57.80200S01’,’ L57.80200S02’,’ |

| | | | |
|---|---|---|---|
| | | 'T78.300' | L57.80200S03’,’ L57.80200S04’,’ L57.80200S05’,’ T78.30000S01’,’ T78.30000S02’,’ T78.30000S03’,’ T78.30000S04’,’ T78.30000S05’. |
| 4 | AC | 'H10.100'，'H10.101'，'H10.102'，'H10.103' | ‘H10.10000S01’,’H10.10100S01’,’ H10.10100S02’,’ H10.10100S03’,’ H10.10100S04’,’ H10.10200S01’,’ H10.10200S02’,’ H10.10300S01’. |
