# Supplementary material for: Characteristics of childhood allergic diseases in outpatient and emergency departments in Shanghai, China, 2016–2018: a multicenter, retrospective study
Source: BMC Pediatr. 2021 Sep 17;21:409. doi: 10.1186/s12887-021-02880-0 (PMC8447671; doi:10.1186/s12887-021-02880-0)
Supplement: Supplementary file 2 — Additional file 2: Supplementary Table 2. Missing rate of gender and distribution comparison of age and payer type between missing group and non-missing group. [file 12887_2021_2880_MOESM2_ESM.pdf]

Supplementary Table 2. Missing rate of gender and distribution comparison of age and payer type between missing group and non-missing group

	Gender Missing Group	Non-missing Group	Missing rate (%)
Asthma, *n*(%)	175	826917	0.0212
Age range, years			
<1	100(57.1)	78989(9.6)	0.1266
1~ <4	54(30.9)	313363(37.9)	0.0172
4~ <7	12(6.9)	268172(32.4)	0.0045
7~ <12	9(5.1)	134371(16.2)	0.0067
12~<=18		32022(3.9)	0.0000
Payer type			
Medical insurance	157(89.7)	539264(65.2)	0.0291
Self-finance	18(10.3)	284987(34.8)	0.0063
AR	1699	543211	0.3128
Age range, years			
<1	52(3.1)	14373(2.6)	0.3618
1~ <4	442(26.0)	133686(24.6)	0.3306
4~ <7	722(42.5)	207631(38.2)	0.3477
7~ <12	384(22.6)	144785(26.7)	0.2652
12~<=18	99(5.8)	42736(7.9)	0.2317
Payer type			
Medical insurance	452(26.6)	349116(64.3)	0.1295
Self-finance	1247(73.4)	194095(36.7)	0.6425
Allergic skin diseases	54	924737	0.0058
Age range, years			
<1	33(61.1)	241955(26.2)	0.0136
1~ <4	14(25.9)	309163(33.4)	0.0045
4~ <7	4(7.4)	170555(18.4)	0.2650
7~ <12	3(5.6)	137207(14.8)	0.0022
12~<=18		65857(7.1)	0.0000
Payer type			
Medical insurance	47(87.0)	492112(53.2)	0.0096
Self-finance	7(13.0)	432625(46.8)	0.0016
AC	6	81285	0.0074
Age range, years			
<1	2	2717(3.3)	0.0736
1~ <4	2	22697(27.9)	0.0088
4~ <7		30045(37.0)	0.0000
7~ <12	2	22881(28.1)	0.0087
12~<=18		2945(3.6)	0.0000
Payer type			

Medical insurance	6	55400(68.2)	0.0108
Self-finance		25885(31.8)	0.0000
