# Supplementary material for: Characteristics of childhood allergic diseases in outpatient and emergency departments in Shanghai, China, 2016–2018: a multicenter, retrospective study
Source: BMC Pediatr. 2021 Sep 17;21:409. doi: 10.1186/s12887-021-02880-0 (PMC8447671; doi:10.1186/s12887-021-02880-0)
Supplement: Supplementary file 3 — Additional file 3: Supplementary Table 3. Comparison of demographic characteristics between outpatient and ED visits. [file 12887_2021_2880_MOESM3_ESM.pdf]

Supplementary Table 3. Comparison of demographic characteristics between outpatient and ED visits

	Characteristic	Total	Outpatient visit	ED visit	ED/Outpatient visits ratio	*P* value
Asthma	Sex					<0.001
	Male	532034(65.6)	422733(65.3)	109301(66.6)	0.26	
	Female	279052(34.4)	224256(34.7)	54796(33.4)	0.24	
	Age range, years					<0.001
	<1	77873(9.6)	65990(10.2)	11883(7.2)	0.18	
	1~ <4	306638(37.8)	243957(37.7)	62681(38.2)	0.26	
	4~ <7	263060(32.4)	212183(32.8)	50877(31.0)	0.24	
	7~ <12	132178(16.3)	100816(15.6)	31362(19.1)	0.31	
	12~<=18	31512(3.9)	24175(3.7)	7337(4.5)	0.30	
	Payer type					<0.001
	Medical insurance	525934(64.8)	415587(64.2)	110347(67.2)	0.27	
	Self-finance	285327(35.2)	231534(35.8)	53793(32.8)	0.23	
AR	Sex					<0.001
	Male	342458(63.3)	328785(63.3)	13673(62.1)	0.41	
	Female	198942(36.7)	190591(36.7)	8351(37.9)	0.44	
	Age range, years					<0.001
	<1	14372(2.6)	13935(2.7)	437(2.0)	0.031	
	1~ <4	133669(24.6)	128954(24.8)	4715(21.2)	0.037	
	4~ <7	207597(38.2)	199642(38.3)	7955(35.8)	0.040	
	7~ <12	144747(26.7)	137652(26.4)	7095(31.9)	0.052	
	12~<=18	42714(7.9)	40678(7.8)	2036(9.2)	0.050	
	Payer type					<0.001
	Medical insurance	349074(64.3)	333650(64.1)	15424(69.4)	0.046	
	Self-finance	194025(36.7)	187211(35.9)	6814(30.6)	0.036	
Allergic skin diseases	Sex					0.092
	Male	513255(55.5)	471996(55.5)	41259(55.2)	0.087	
	Female	411428(44.5)	377958(44.5)	33470(44.8)	0.089	
	Age range, years					<0.001
	<1	241955(26.2)	228501(26.8)	13454(18.0)	0.059	
	1~ <4	309163(33.4)	282031(33.2)	27132(36.3)	0.096	
	4~ <7	170555(18.4)	154417(18.2)	16138(21.6)	0.105	
	7~ <12	137207(14.8)	125108(14.7)	12099(16.2)	0.097	
	12~<=18	65857(7.1)	59948(7.1)	5909(7.9)	0.099	

	Payer type					<0.001
	Medical insurance	492112(53.2)	447830(52.7)	44282(59.3)	0.099	
	Self-finance	432625(46.8)	402175(47.3)	30450(40.7)	0.076	
AC	Sex					0.872
	Male	52587(64.7)	51506(64.7)	1081(64.5)	0.021	
	Female	28672(35.3)	28077(35.3)	595(35.5)	0.021	
	Age range, years					<0.001
	<1	2717(3.3)	2700(3.4)	17(1.0)	0.006	
	1~ <4	22694(27.9)	22406(28.2)	288(17.2)	0.013	
	4~ <7	30039(37.0)	29408(36.9)	631(37.6)	0.021	
	7~ <12	22873(28.1)	22281(28.0)	592(35.3)	0.027	
	12~<=18	2942(3.6)	2794(3.5)	148(8.8)	0.053	
	Payer type					0.089
	Medical insurance	55390(68.2)	54215(68.1)	1175(70.1)	0.022	
	Self-finance	25875(31.8)	25374(31.9)	501(29.9)	0.020	
